# Analysis of the burden of intracerebral hemorrhage in the Asian population aged 45 and older and ARIMA model prediction trends: a systematic study based on the GBD 2021

**DOI:** 10.3389/fneur.2025.1526524

**Published:** 2025-02-13

**Authors:** Minghong Xu, Chao Tang, Yongkai Shen, Yinan Zhang, Long Bao

**Affiliations:** ^1^Department of Neurosurgery, The First Affiliated Hospital of Jinzhou Medical University, Jinzhou, China; ^2^Department of Neurosurgery, General Hospital of Fushun Mining Bureau of Liaoning Health Industry Group, Fushun, China

**Keywords:** intracerebral hemorrhage (ICH), The Global Burden of Disease (GBD) Study, age-standardized incidence rate (ASIR), age-standardized mortality rate (ASMR), age-standardized disability-adjusted life-year rate (ASDR)

## Abstract

**Background:**

Intracerebral hemorrhage (ICH), a severe subtype of hemorrhagic stroke, is associated with significant disability and high mortality rates. Due to population aging and the prevalence of hypertension in the Asian region, intracerebral hemorrhage has become one of the major causes of high disability and mortality. This study analyzes the epidemiological patterns of ICH across Asia from 1990 to 2021 and projects potential trends for the period 2022 to 2041.

**Methods:**

This study extracted four key indicators related to intracerebral hemorrhage (ICH) from The Global Burden of Disease (GBD) 2021 database for the years 1990 to 2021: prevalence, incidence, mortality, and disability-adjusted life years (DALYs). The age-period-cohort model was employed to assess the impact of age, time periods, and birth cohorts on ICH trends. Additionally, the autoregressive integrated moving average (ARIMA) model was utilized to conduct long-term trend analysis and forecast the changing trends of various indicators from 2022 to 2041.

**Results:**

From 1990 to 2021, age-standardized incidence rate (ASIR), age-standardized mortality rate (ASMR), and age-standardized disability-adjusted life-year rate (ASDR) of ICH in Asia exhibited an overall declining trend, the ASIR declined from 82.35 per 100,000 (95% UI: 70.73–93.35) to 52.35 per 100,000 (95% UI: 45.98–58.46). Similarly, the ASMR dropped from 92.02 per 100,000 (95% UI: 83.06–101.24) to 53.26 per 100,000 (95% UI: 47.61–58.96), while the ASDR fell from 2,094.51 per 100,000 (95% UI: 1,916.68–2,293.61) to 1,194.11 per 100,000 (95% UI: 1,072.05–1,306.04). The age effect demonstrated that the relative risk (RR) of ICH increases with age, peaking in the 90–94 age group. The period effect indicated that the risk did not increase over time, while the cohort effect suggested a declining trend in later-born cohorts. The ARIMA model’s predictions indicate that over the next 20 years, the age-standardized rates in Asia, except for prevalence, will generally show a declining trend.

**Conclusion:**

The disease burden of ICH in Asia varies by gender and age group. According to ARIMA model predictions, while the overall burden of ICH is expected to decline over the next 20 years, the age-standardized prevalence rate is projected to increase due to population aging. Given the high mortality and disability rates associated with ICH, its disease burden remains significant and requires focused attention. Strengthening screening and hypertension management in high-risk elderly populations, along with community health education and early intervention, is recommended to reduce the risk of ICH.

## 1 Introduction

Intracerebral hemorrhage (ICH) is a critical subtype of stroke characterized by bleeding into the brain parenchyma, often resulting in severe disability or death (1–3). Although ICH accounts for only 10–20% of all strokes globally, it disproportionately contributes to stroke-related mortality and long-term neurological impairment (4). Asia bears a particularly high burden of ICH due to its large, aging population and prevalent risk factors such as hypertension (5), diabetes, and lifestyle-related behaviors (6). Compared to Western populations, the incidence of ICH in Asia is significantly higher, posing considerable public health challenges (2, 7). Understanding the trends in ICH burden is essential for effective public health planning and resource allocation, especially in the context of rapid demographic shifts toward an aging population.

The Global Burden of Disease (GBD) Study, a comprehensive epidemiological initiative, offers valuable insights into global disease trends by systematically analyzing a wide range of diseases and risk factors (8, 9). The 2021 GBD Study provides a unique opportunity to assess the burden of ICH in Asia, using key metrics such as incidence, prevalence, mortality, and disability-adjusted life years (DALYs) (10, 11). Extensive data from 1990 to 2021 can be utilized to evaluate the current state of ICH in Asia and project future trends, which is particularly important given the region’s rapid demographic changes (12).

As the population in many Asian countries continues to age, understanding the evolution of the intracerebral hemorrhage burden over the past three decades and identifying patterns influenced by age, period, and cohort effects have become increasingly important. While previous studies have highlighted the significant impact of intracerebral hemorrhage on Asian populations (13), there is still a lack of detailed longitudinal analyses focusing on age-specific trends and projections. This study leverages the 2021 Global Burden of Disease database to assess changes in four key indicators of intracerebral hemorrhage among Asians aged 45 and older from 1990 to 2021, employing an innovative combination of APC and ARIMA models to forecast future burden trends.

Considering the complex interplay of demographic shifts, healthcare access, and lifestyle changes in Asia, a cautious approach is essential when interpreting the trends and projections in this study. Our goal is to elucidate the shifting landscape of the ICH burden in Asia, thereby contributing to evidence-based public health strategies aimed at mitigating the impact of this debilitating condition.

## 2 Methods

### 2.1 Data source

The GBD 2021 database encompasses over 370 diseases and injuries across 204 countries and regions, providing comprehensive data on incidence, prevalence, mortality, and age-standardized rates (ASR) (14). To ensure data quality, information is meticulously extracted from reputable public databases after thorough screening. The GBD team updates the data annually to maintain accuracy (10). Data collected from various networks by GBD collaborators undergo rigorous cleaning, transformation, and modeling, with estimates produced by research institutions worldwide (15). Sophisticated methods address any missing data, with adjustments made for confounding factors. Detailed descriptions of the study design and methodologies used in GBD research are extensively documented in existing literature (10).

### 2.2 Definition of ICH

The ICH occurs when a blood vessel ruptures, leading to bleeding within the brain. It is a leading cause of death and long-term disability worldwide. The condition is defined according to the standards of the International Classification of Diseases (16), 10th Revision (ICD-10).

### 2.3 Estimation framework

The GBD employs complex statistical models to estimate incidence, prevalence, and mortality rates. Its unique model, DisMod-MR (Disease Modeling Meta-Regression) (17), integrates various data sources through meta-regression analysis to generate estimates across different populations and regions. This Bayesian geospatial software synthesizes various disease parameters, epidemiological models, and spatial data to generate precise, reliable estimates (10). The 95% uncertainty interval (95%UI) is used to account for variability that may arise from model assumptions (18), helping to convey the reliability of the estimate and the related uncertainty. Age-standardized rates (ASRs) enable fair comparisons of disease burden across regions and populations by adjusting for age distribution differences (16).

### 2.4 Age-period-cohort model (APC model)

The study utilizes the APC model (19) to assess how variations in age, time periods, and birth cohorts influence changes in incidence rates. In this model, the age effect captures the impact of different age groups on incidence rates, while the period effect reflects influences affecting all age groups within a specific time frame, potentially linked to economic, social, or other changes. The cohort effect highlights unique health risks faced by individuals born in a specific era. For accurate analysis, the APC model requires equal intervals across age, period, and cohort categories; otherwise, overlapping data may distort results.

### 2.5 ARIMA model predicts four age-standardized rates

The ARIMA model was employed to forecast trends in four age-standardized indicators of ICH over the next 20 years (20). This model effectively captures patterns and seasonal fluctuations in time series data by integrating three key components: autoregression (AR), differencing (I), and moving average (MA). Its parameters, denoted as p, d, and q, represent the autoregressive order, the degree of differencing, and the moving average order, respectively (21). Stationarity was assessed using the Augmented Dickey-Fuller (ADF) test, while the autocorrelation function (ACF) and partial autocorrelation function (PACF) were used to determine optimal values for p and q.

The optimal ARIMA models were selected using the Akaike Information Criterion (AIC) and Bayesian Information Criterion (BIC) to predict the ICH disease burden from 2021 to 2041. The Ljung–Box *Q* test confirmed that the residuals of the chosen models followed an independent normal distribution. All analyses, except for those involving the APC model (which used the National Cancer Institute’s online tool), were performed using the R statistical software (version 4.4.1).

## 3 Results

### 3.1 Descriptive analysis

**Figure 1:**
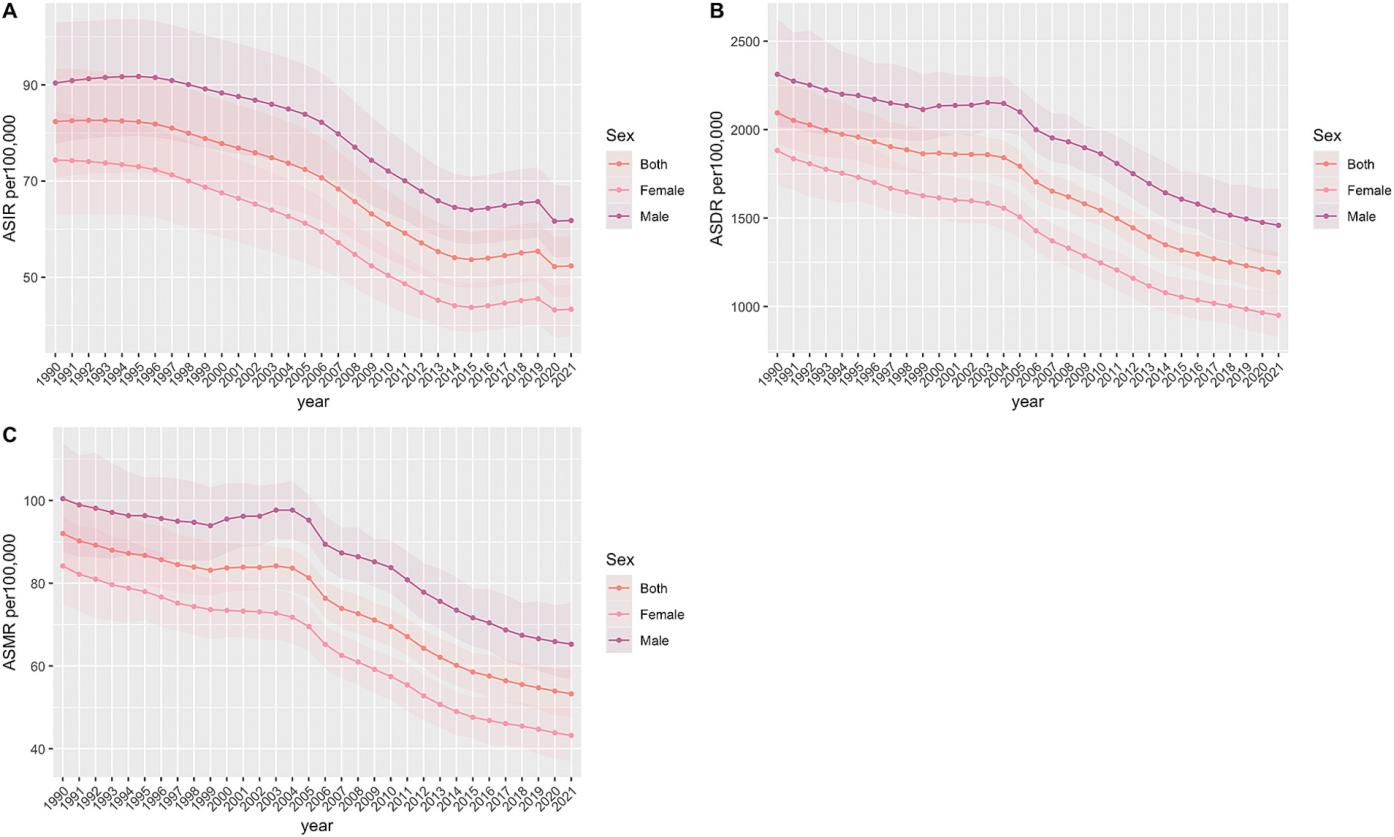
Age-standardized incidence, DALY and death rate of intracerebral hemorrhage per 100,000 population from 1990 to 2021. **(A)** Age-standardized incident rate. **(B)** Age-standardized DALY rate. **(C)** Age-standardized death rate.

The trends in age-standardized incidence rate (ASIR), age-standardized mortality rate (ASMR), and age-standardized disability-adjusted life-year rate (ASDR) for ICH in Asia from 1990 to 2021 are shown in Figure 1 and Table 1.

**Table 1:** Asia age-standardized rate of incidence, deaths, and DALYs of Intracerebral hemorrhage (ICH) from 1990 to 2021.

Year	Measure/100,000 persons (95% UI)	Male	Female	Both
1990	ASIR	90.36 (77.71, 102.92)	74.35 (63.1, 84.53)	82.35 (70.73, 93.35)
	ASMR	100.44 (87.47, 113.88)	84.16 (75.00, 96.02)	92.02 (83.06, 101.24)
	ASDR	2312.35 (2016.01, 2625.66)	1881.45 (1685.31, 2107.1)	2094.51 (1916.68, 2293.61)
2021	ASIR	61.79 (54.26, 68.95)	43.34 (37.64, 48.43)	52.35 (45.98, 58.46)
	ASMR	65.26 (56.74, 75.17)	43.19 (36.97, 49.77)	53.26 (47.61, 58.96)
	ASDR	1458.62 (1283.25, 1665.52)	950.4 (827.11, 1080.45)	1194.11 (1072.05, 1306.04)

ASIR, age-standardized incidence rate; ASMR, age-standardized mortality rate; ASDR, age-standardized disability-adjusted life-year rate; UI, uncertainty interval.

From 1990 to 2021, ASIR for ICH exhibited fluctuations. There was a gradual increase from 1990 to 1995, followed by a slow decline that accelerated between 1995 and 2015. This was succeeded by an upward trend from 2015 to 2019, and finally, a decline that stabilized between 2019 and 2021. In contrast, ASDR showed a more complex pattern: an initial decline, a subsequent increase, and then another decline. Specifically, the ASDR experienced a slow decrease from 1990 to 1999, an upward trend from 1999 to 2003, and a consistent decrease from 2003 to 2021.

Table 1 presents ASIR, ASMR, and ASDR for ICH in Asia, stratified by gender for the years 1990 and 2021. From 1990 to 2021, the ASIR per 100,000 population decreased for both genders: in males, it declined from 90.36 (95% UI: 77.71–102.92) to 61.79 (95% UI: 54.26–68.95), and in females, from 74.35 (95% UI: 63.10–84.53) to 43.34 (95% UI: 37.64–48.43). The ASMR for males dropped from 100.44 (95% UI: 87.47–113.88) to 65.26 (95% UI: 56.74–75.17), and for females, from 84.16 (95% UI: 75.00–96.02) to 43.19 (95% UI: 36.97–49.77). Similarly, the ASDR for males decreased from 2312.35 (95% UI: 2016.01–2625.66) to 1458.62 (95% UI: 1283.25–1665.52), and for females, from 1881.45 (95% UI: 1685.31–2107.10) to 950.40 (95% UI: 827.11–1080.45).

**Figure 2:**
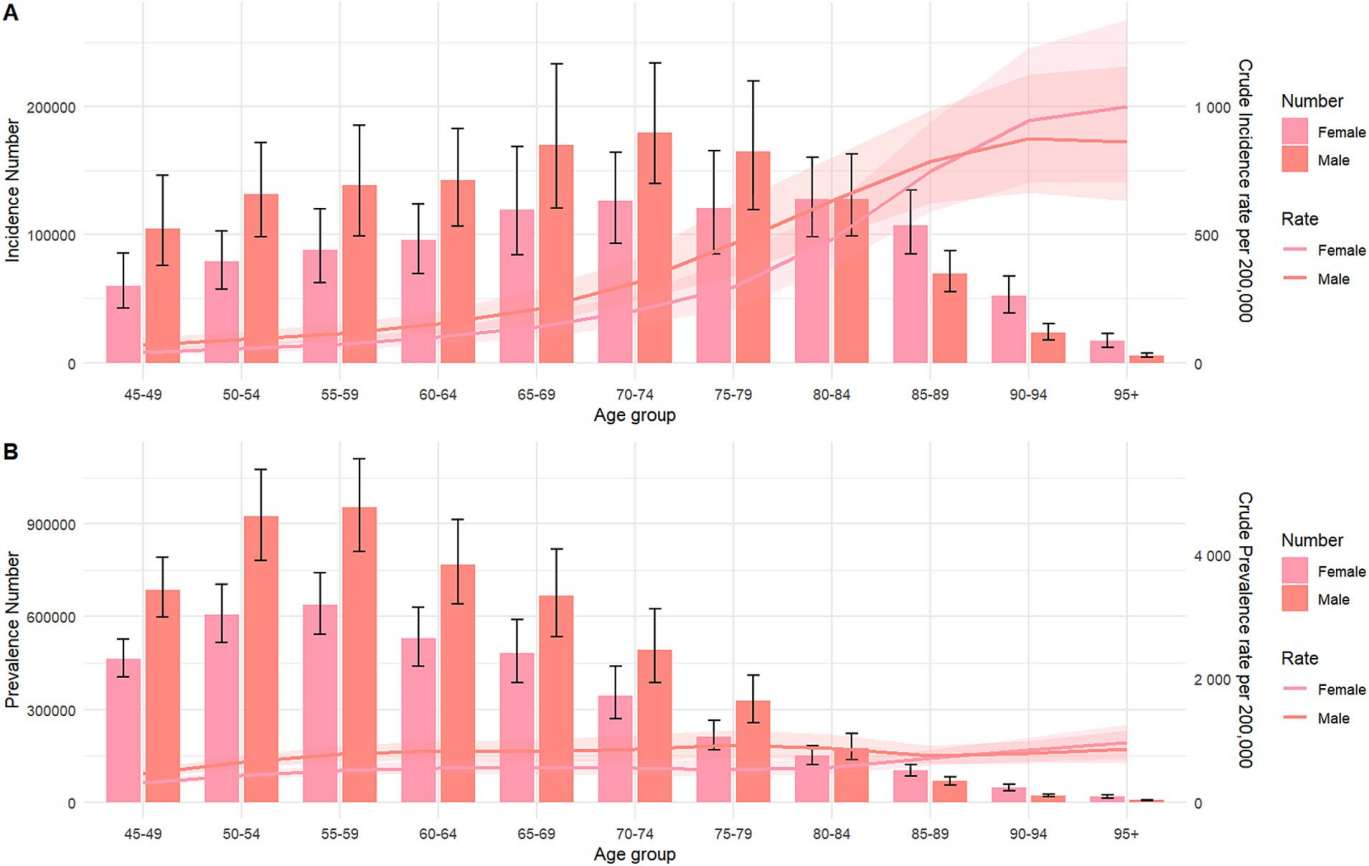
Number of incidence, prevalence cases and incidence, prevalence rate of intracerebral hemorrhage per 100,000 population, by age and sex in 2021. Lines indicate prevalent case with 95% uncertainty intervals for male and female. **(A)** Incidence; **(B)** prevalence.

Supplementary Table S1 and Supplementary Figures S1, S2 present gender-specific data on the incidence, mortality, prevalence, and disability-adjusted life years (DALYs) for ICH in Asia, stratified by age group from 1990 to 2021. The highest prevalence rate was observed in males aged 95 and older, whereas for females, it peaked in the 75–79 age group. The incidence rate reached its maximum in the 70–74 age group for both genders. Mortality rates were highest in males aged 70–74 and females aged 75–79. The DALYs rate was greatest in the 65–69 age group for both genders. Figure 2 illustrates the relationship between the number of cases and the incidence and prevalence rates, stratified by age group for the year 2021.

To compare the burden of ICH across Asian regions, we analyzed the age-standardized rates of four ICH indicators for China, India, Singapore, and Japan in 2021. As indicated in Table 2, China recorded the highest ASIR, ASMR, and ASDR, while Japan had the highest age-standardized prevalence rate (ASPR).

**Table 2:** Age-standardized incidence rate (ASIR), ASMR, ASDR, ASPR in China, India, Japan, and Singapore in 2021.

Measure/100,000 Persons (95% UI)	China	India	Singapore	Japan
ASIR	61.15 (52.98, 69.06)	43.85 (37.43, 50.48)	17.18 (15.06, 19.23)	19.12 (16.65, 21.63)
ASMR	68.84 (57.61, 81.17)	35.43 (28.42, 41.45)	5.94 (5.32, 6.44)	10.01 (8.76, 10.76)
ASDR	1351.55 (1129.11, 1600.86)	888.62 (733.24, 1030.81)	165.73 (151.42, 179.62)	251.76 (231.92, 268.19)
ASPR	222.11 (200.09, 246.48)	198.75 (177.19, 223.67)	210.34 (199.57, 221.69)	222.85 (200.39, 246.82)

ASIR, age-standardized incidence rate; ASMR, age-standardized mortality rate; ASDR, age-standardized disability-adjusted life-year rate; ASPR, age-standardized prevalence rate; UI, uncertainty interval.

### 3.2 Age-period-cohort analysis

**Figure 3:**
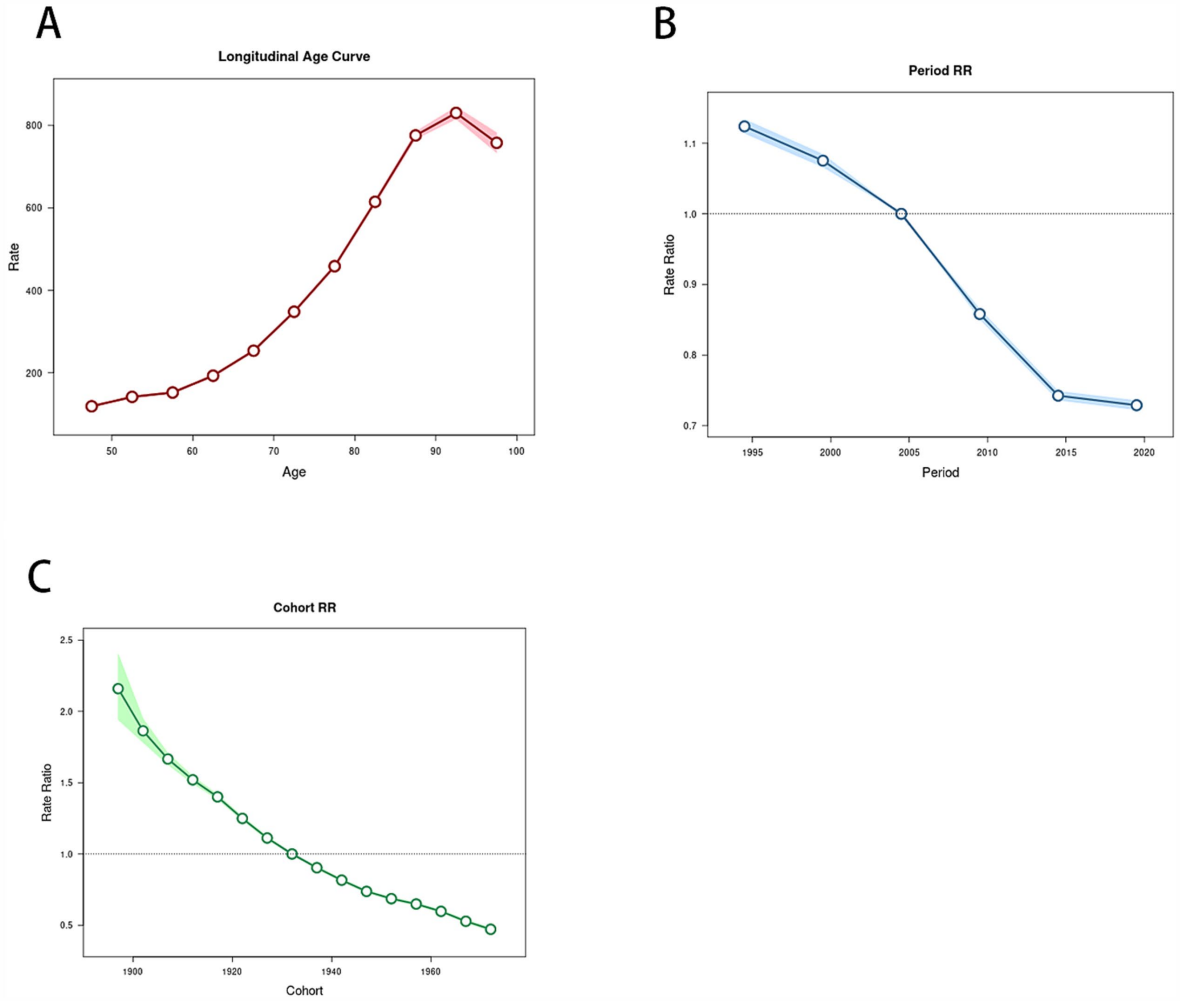
Relative risks of the incidence of intracerebral hemorrhage in Asia from 1990 to 2021 due to effects of age, period, and cohort. **(A)** Age effects on intracerebral hemorrhage; **(B)** period effects on intracerebral hemorrhage; **(C)** cohort effects on intracerebral hemorrhage.

Supplementary Table S2 and Figure 3 present the results of the age, period, and cohort effects on ICH. The net drift, calculated at −1.94% per year (95% CI: −1.974 to −1.906), indicates that from 1992 to 2021, the average annual incidence or mortality rate decreased by 1.94%. The Wald tests, using the Chi-square test, assessed the impact of various factors, revealing significant differences across all age groups (*p* < 0.001), periods (*p* < 0.001), and cohorts (*p* < 0.001). Figure 3A displays the incidence rates across age groups, with the highest rate in the 90–94 age group. Figure 3B illustrates the period effects, indicating a decline in the relative risk (RR) of ICH incidence, which slowed after 2014. Figure 3C highlights the cohort effects, demonstrating a continuous decrease in the RR of ICH incidence among later-born cohorts.

### 3.3 The ARIMA model predicts four age-standardized rates

**Figure 4:**
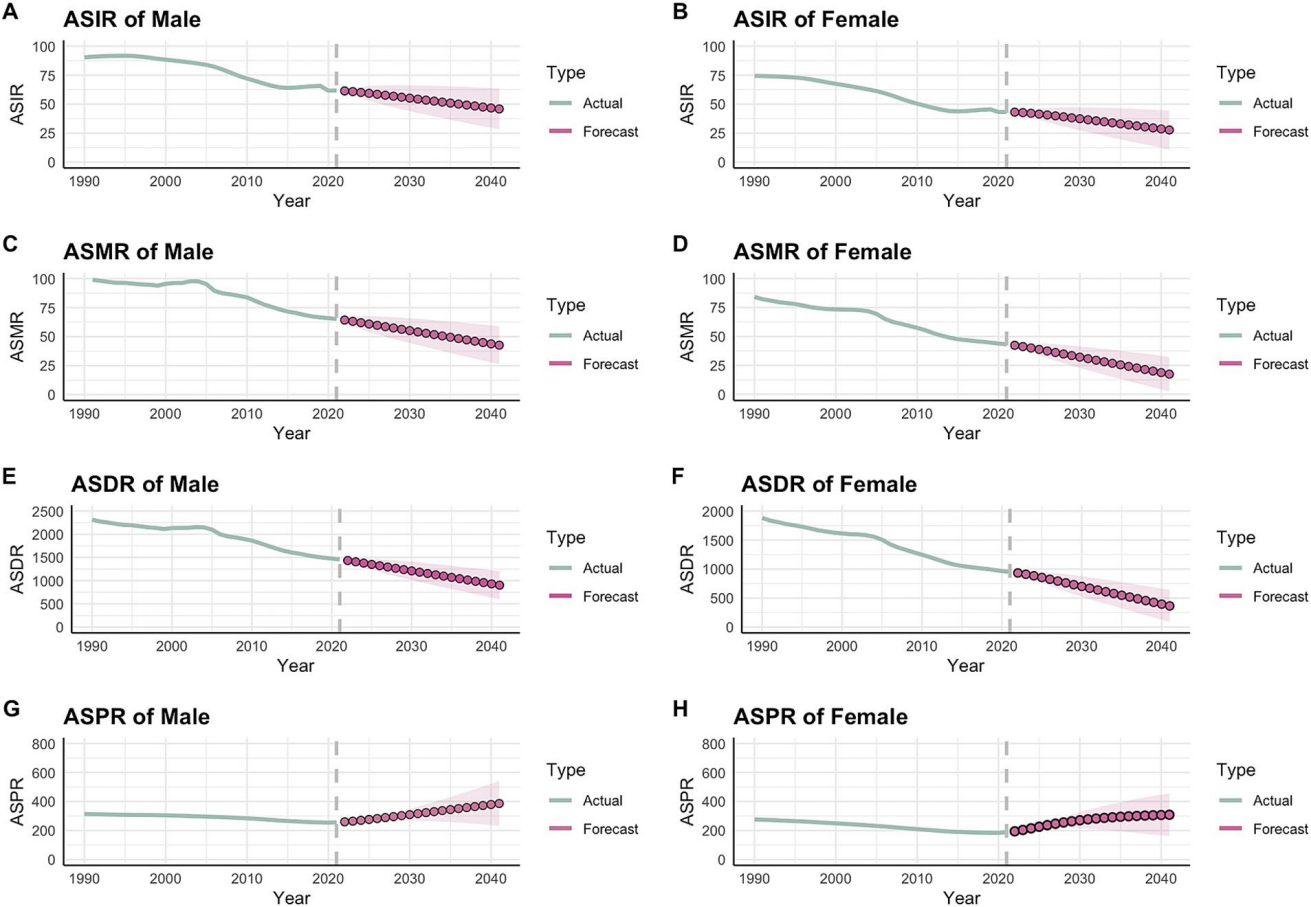
Forecast of intracerebral hemorrhage ASIR, ASMR, ASDR, and ASPR per 100,000 from 2022 to 2041 through ARIMA. **(A)** ASIR of Male; **(B)** ASIR of Female; **(C)** ASMR of Male; **(D)** ASMR of Female; **(E)** ASDR of Male; **(F)** ASDR of Female; **(G)** ASPR of Male; **(H)** ASPR of Female.

The ARIMA model was employed to forecast trends in four age-standardized indicators of ICH over the next 20 years. The optimal model parameters, along with their corresponding AIC, BIC, and Ljung–Box test *p*-values, are detailed in Table 3. The Ljung–Box test confirmed that all models exhibited white noise residuals, demonstrating their stability and indicating a strong fit to the data. Figure 4 illustrates the projected trends in age-standardized rates from 2022 to 2041.

**Table 3:** Autoregressive integrated moving average (ARIMA) model parameters and their corresponding AIC and BIC for prediction of ASIR, ASMR, ASDR and ASPR (per 100,000) for ICH for the next 20 years in Asia.

Measures	Sex	Parameters	AIC	BIC	Ljung–Box test *p*-value
Incidence	Male	ARIMA (1,1,0)	94.48	98.78	0.9931
	Female	ARIMA (1,1,0)	68.91	73.21	0.9869
Deaths	Male	ARIMA (0,1,1)	100.95	105.25	0.9280
	Female	ARIMA (1,1,1)	63.39	69.12	0.9928
DALYs	Male	ARIMA (0,1,1)	273.83	278.13	0.6935
	Female	ARIMA (2,1,0)	236.17	241.9	0.9787
Prevalence	Male	ARIMA (1,2,0)	57.11	59.92	0.9735
	Female	ARIMA (2,1,2)	86.44	95.04	0.9998

In contrast to the declining trends in age-standardized incidence, mortality, and DALYs for both men and women with ICH from 2022 to 2041, the projected ASPR is expected to increase by 2041. Specifically, the projected age-standardized rates for males are as follows: incidence at 45.88 per 100,000, mortality at 42.63 per 100,000, prevalence at 902.83 per 100,000, and DALYs at 386.18 per 100,000. For females, the corresponding rates are: incidence at 27.78 per 100,000, mortality at 17.41 per 100,000, prevalence at 365.17 per 100,000, and DALYs at 308.52 per 100,000. Supplementary Table S3 provides the detailed yearly projections from 2022 to 2041.

## 4 Discussion

To the best of our knowledge, this is the first study utilizing GBD 2021 data to analyze long-term trends in ICH incidence in Asia from 1990 to 2021 using age-period-cohort models, while also applying the ARIMA model to forecast changes in age-standardized rates of ICH in the region.

The results indicated that from 1990 to 2021, the incidence, prevalence, mortality, and DALYs of ICH in Asia demonstrated a declining trend. However, ARIMA model projections for the next 20 years revealed that while incidence, mortality, and DALYs will continue to decrease, prevalence is expected to rise. Additionally, susceptibility increases with age, with males being more vulnerable than females. These findings can guide policymakers in Asia to develop targeted strategies and inform future research. According to a recent GBD article (6) analyzing the global, regional, and national burden of ICH found that trends in prevalence, mortality, and DALYs in Asia align with global patterns, all showing a decline. In 2021, the global age-standardized prevalence rate of ICH was 40.8 per 100,000 population, reflecting a 31.4% decrease from 1990. The mortality rate stood at 39.1 per 100,000, a reduction of 36.6%, while the DALYs rate was 92.4 per 100,000, down by 39.1%.

This downward trend may be attributed to several factors. Firstly, economic development, societal progress, and increasing levels of education have made health-related information more accessible. As a result, people are placing greater emphasis on maintaining their health, particularly through a balanced diet, which may help control key risk factors for ICH, such as hypertension, hyperlipidemia, and high BMI (22, 23). Additionally, improved access to exercise facilities has made it easier for individuals to engage in regular physical activity, which can indirectly lower blood pressure, cholesterol levels, and body fat. Secondly, advancements in medical imaging, particularly the widespread use of CT scans, have significantly enhanced the diagnosis of acute intracerebral hemorrhage. Today, CT is often the preferred initial imaging technique due to its convenience, cost-effectiveness, and high sensitivity in detecting ICH. It enables clinicians to quickly identify the location and estimate the extent of bleeding, thus facilitating more accurate diagnosis and timely treatment planning.

Currently, two primary surgical approaches are used to treat ICH: traditional craniotomy and minimally invasive procedures, such as endoscopic surgery (ES) and stereotactic aspiration (SA). Studies have shown that ES and SA are gaining acceptance because they involve smaller incisions and cause less damage to brain tissue (24). A meta-analysis of 2,105 patients (705 in the ES group and 1,400 in the SA group) revealed that ES outperformed SA in achieving better mortality rates, improved functional outcomes, and greater hematoma clearance within 1 day post-surgery, leading to reduced mortality and residual hematoma. However, SA demonstrated advantages over ES in reducing perihematoma edema, shortening surgery duration, minimizing intraoperative blood loss, and decreasing hospital stay lengths (25). This suggests that ongoing advancements and refinements in surgical techniques are closely linked to reduced mortality and decreased DALYs in patients with ICH.

The forecast suggests that population aging in Asia (26) over the next 20 years may be closely associated with the increase in ASPR, potentially offsetting the decline in ASIR. Therefore, we must remain vigilant. When formulating future public health policies, the focus should be on high-risk populations and countries. A series of measures should be implemented to improve hypertension management, control smoking, enhance sanitation (27, 28), and expand the coverage of public healthcare services to reduce the age-standardized rate of ICH. These efforts aim to alleviate the disease burden of ICH in Asia. This study reveals that men have higher incidence, prevalence, DALYs, and mortality rates than women (29), likely due to lifestyle factors, dietary habits, and notably, higher smoking rates (30, 31) among men. Given that Asia is the most populous continent, the burden of ICH is particularly significant. Although morbidity, mortality, and DALYs have generally decreased since 1990, the incidence, mortality, and prevalence of ICH continue to rise with advancing age, placing a substantial strain on society.

Considering these challenges, it is essential to develop practical strategies to alleviate the disease burden of ICH and allocate adequate medical resources to high-risk groups. Effective interventions are needed to mitigate the impact of ICH on both national healthcare systems and society as a whole.

This study possesses several strengths. To our knowledge, it is the first to comprehensively analyze the incidence, prevalence, mortality, and DALYs of ICH by sex and age group in Asia, while also making projections of the disease burden over the next 20 years. The methodologies applied have proven robust in previous GBD studies. Additionally, GBD data are analyzed using standardized techniques, ensuring that these estimates are consistent and comparable over time. Furthermore, this study is the first to integrate APC analysis with the ARIMA model to assess the temporal impact on ICH incidence in Asia and predict the region’s age-standardized ICH rate, offering valuable insights for future prevention and control strategies.

However, it is undeniable that this study has certain limitations. Some issues from the GBD 2019 study persist, such as potential gaps in data quality and coverage in certain regions or countries. Additionally, the assumptions underlying statistical models may fail to fully capture the complexities of real-world scenarios. Furthermore, our disease forecasts are based on historical trends, which may limit the accuracy of predictions, particularly in accounting for unforeseen crises or sudden shifts in the future. Future research should further evaluate the effectiveness of interventions across different countries and regions, and develop new predictive models to improve accuracy.

## 5 Conclusion

This study offers valuable data on ICH that hold significant public health implications for Asia. To mitigate the impact of ICH on countries, societies, and individuals, it is essential to develop targeted intervention policies aimed at high-risk populations, with a focus on prevention and treatment. As the aging population continues to grow rapidly, greater attention must be directed toward addressing this critical issue.

## Data Availability

The original contributions presented in the study are included in the article/Supplementary material, further inquiries can be directed to the corresponding author.

## Glossary

ASDR: Age-standardized disability-adjusted life-year rate
ASIR: Age-standardized incidence rate
ASMR: Age-standardized mortality rate
ASPR: Age-standardized prevalence rate
ASR: Age-standardized rate
GBD: The Global Burden of Disease Study
ICD: International Classification of Disease
UI: Uncertainty interval
ICH: Intracerebral hemorrhage
DALYs: Disability-adjusted life-years
ARIMA: Autoregressive integrated moving average
ADF: Augmented Dickey–Fuller
ACF: Autocorrelation function
PACF: Partial autocorrelation function
AIC: Akaike information criterion
BIC: Bayesian information criterion
APC: Age-Period-Cohort model
