# Supplementary material for: Analysis of the burden of intracerebral hemorrhage in the Asian population aged 45 and older and ARIMA model prediction trends: a systematic study based on the GBD 2021
Source: Front Neurol. 2025 Feb 13;16:1526524. doi: 10.3389/fneur.2025.1526524 (PMC11869383; doi:10.3389/fneur.2025.1526524)
Supplement: Supplementary file 1 [file Table_1.DOCX]

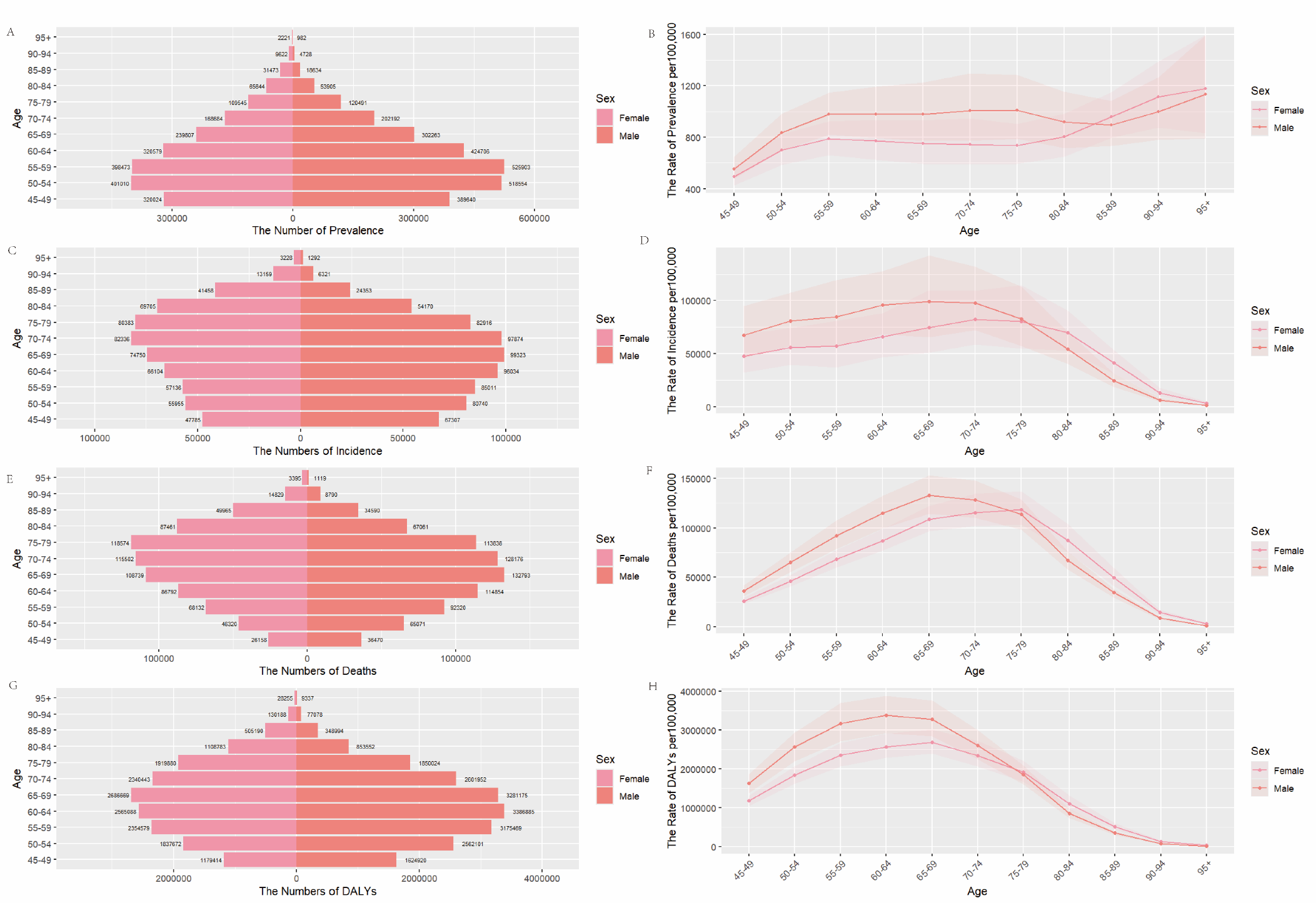


**Figure S1** The numbers of incidence, prevalence, deaths, DALYs, as well as the incidence rate, prevalence rate, mortality rate, and DALYs rate by sex and age groups in Asia in 1990


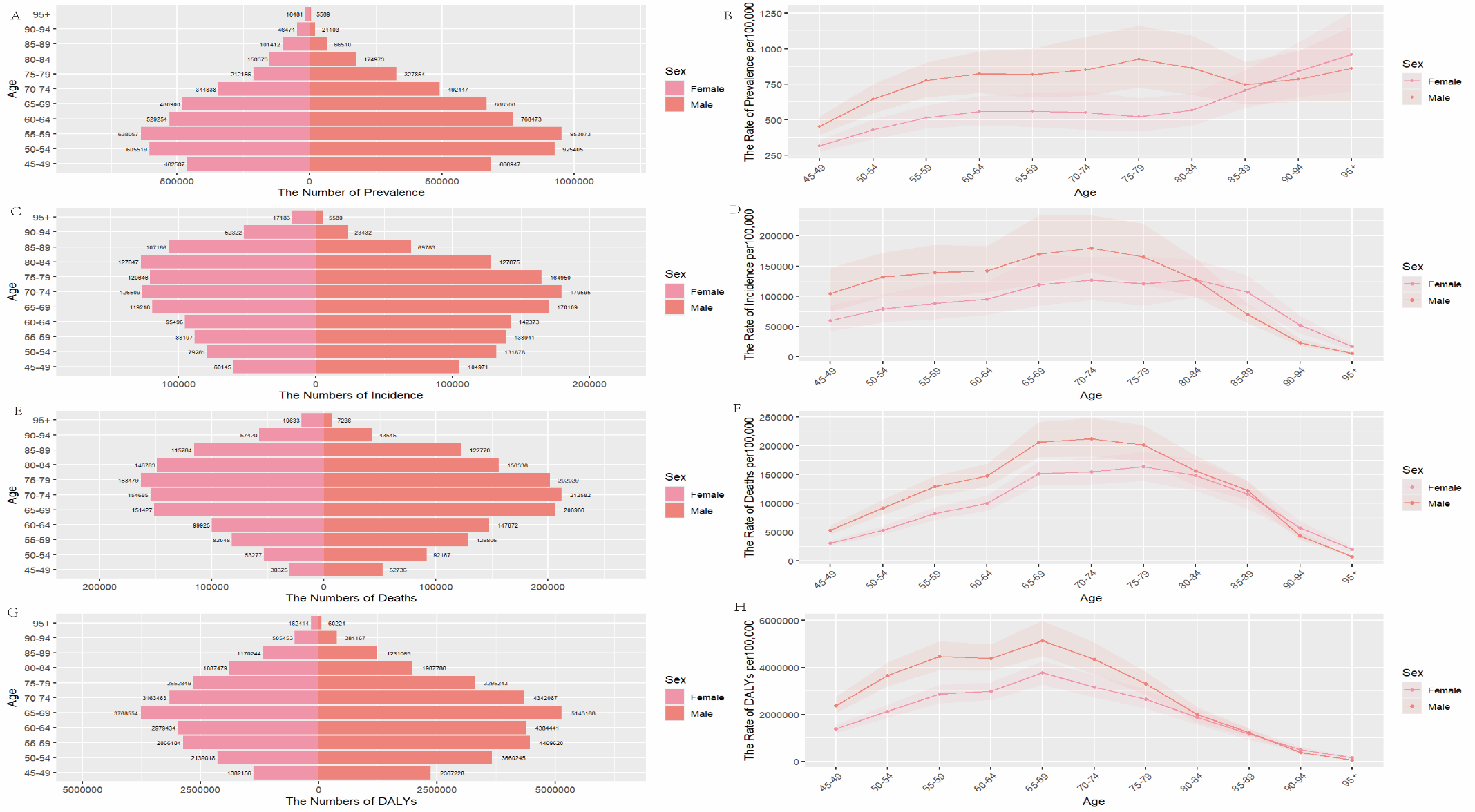


**Figure S2** The numbers of incidence, prevalence, deaths, DALYs, as well as the incidence rate, prevalence rate, mortality rate, and DALYs rate by sex and age groups in Asia in 2021
